# miR-34a/DRP-1-mediated mitophagy participated in cisplatin-induced ototoxicity via increasing oxidative stress

**DOI:** 10.1186/s40360-023-00654-1

**Published:** 2023-03-07

**Authors:** Haiyan Wang, Hanqing Lin, Weibiao Kang, Lingfei Huang, Sisi Gong, Tao Zhang, Xiaotong Huang, Feinan He, Yongyi Ye, Yiyang Tang, Haiying Jia, Haidi Yang

**Affiliations:** 1 Department of Otolaryngology, the First Affiliated Hospital of Jinan University, No.613, West Huangpu Avenue, Guangzhou, 510630 China; 2 Department of Otolaryngology, the 2nd hospital, Medical College, Shantou University, Shantou, Guangdong China; 3 Department of Otolaryngology, Sun Yat-Sen Memorial Hospital, Sun Yat-Sen University, 107 West Yan Jiang Road, Guangzhou, 510120 China; 4 Guangdong Provincial Key Laboratory of Malignant Tumor Epigenetics and Gene Regulation, Medical Research Center, Sun Yat-Sen Memorial Hospital, Sun Yat-Sen University, Guangzhou, China; 5 Department of Hearing and Speech Science, Xinhua College, Sun Yat-Sen University, Guangzhou, China

**Keywords:** Ototoxicity, miR-34a, DRP-1, Mitophagy, Cisplatin

## Abstract

**Purpose:**

Cisplatin is a widely used and effective chemotherapeutic agent for most solid malignant tumors. However, cisplatin-induced ototoxicity is a common adverse effect that limits the therapeutic efficacy of tumors in the clinic. To date, the specific mechanism of ototoxicity has not been fully elucidated, and the management of cisplatin-induced ototoxicity is also an urgent challenge. Recently, some authors believed that miR34a and mitophagy played a role in age-related and drug-induced hearing loss. Our study aimed to explore the involvement of miR-34a/DRP-1-mediated mitophagy in cisplatin-induced ototoxicity.

**Methods:**

In this study, C57BL/6 mice and HEI-OC1 cells were treated with cisplatin. MiR-34a and DRP-1 levels were analyzed by qRT‒PCR and western blotting, and mitochondrial function was assessed via oxidative stress, JC-1 and ATP content. Subsequently, we detected DRP-1 levels and observed mitochondrial function by modulating miR-34a expression in HEI-OC1 cells to determine the effect of miR-34a on DRP-1-mediated mitophagy.

**Results:**

MiR-34a expression increased and DRP-1 levels decreased in C57BL/6 mice and HEI-OC1 cells treated with cisplatin, and mitochondrial dysfunction was involved in this process. Furthermore, the miR-34a mimic decreased DRP-1 expression, enhanced cisplatin-induced ototoxicity and aggravated mitochondrial dysfunction. We further verified that the miR-34a inhibitor increased DRP-1 expression, partially protected against cisplatin-induced ototoxicity and improved mitochondrial function.

**Conclusion:**

MiR-34a/DRP-1-mediated mitophagy was related to cisplatin-induced ototoxicity and might be a novel target for investigating the treatment and protection of cisplatin-induced ototoxicity.

## Introduction

Cisplatin is one of the earliest approved platinum compounds and is a widely used and highly effective chemotherapeutic medicine for many types of tumors including ovarian, uterine, testicular malignant tumors, head and neck cancer and other solid tumors [1, 2], greatly improve the survival rate and quality of life of tumor patients. However, it was reported that the incidence of ototoxicity is approximately 40%-80% [3, 4]. Cisplatin causes bilateral, progressive and irreversible sensorineural hearing loss, often associated with vertigo and tinnitus [3]. To date, there is a lack of available prevention and treatment for ototoxicity. Therefore, ototoxicity limits the maximum treatment effect of tumors and negatively affects the quality life and long-term survival of tumor survivors, particularly children and adolescents with cancer [5]. To date, the precise molecular mechanism of cisplatin-induced ototoxicity has been incompletely elucidated. Thus, further elucidating the pathogenesis of cisplatin-induced ototoxicity is an important research objective for developing a novel therapy. At present, it is widely accepted that the accumulation of reactive oxygen species (ROS) in the cochlea plays an important role in the process, possibly involving inflammation, apoptosis, pyroptosis, ER stress, autophagy and necroptosis [6, 7]. Hearing loss results at least in part from excessive ROS generation in cochlear cells, leading to mitochondrial damage, metabolic disruption, and cell death [4].

Therefore, it is necessary to further investigate the mechanism of cisplatin-induced ototoxicity.

MicroRNAs (miRNAs) are endogenous RNAs of approximately 22 nt that can play important regulatory roles in animals [8]. They regulate gene and protein expression by binding to target mRNA, leading to mRNA degradation or inhibition of translation [9]. Therefore, they have a negative regulatory effect on the relevant gene or protein expression. MiRs are involved in multiple cellular processes, such as development, differentiation, proliferation, autophagy, mitophagy and apoptosis [10, 11].

Recently, miRs were observed to be highly expressed in various cells of the animal cochlea and associated with inner ear development and pathogenesis [12, 13]. MiR-34a is involved in senescence, apoptosis, autophagy and cell death [14, 15]. Previous studies have suggested that miR-34a plays an important role in acquired sensorineural hearing loss, such as age-related hearing loss [8, 12, 16] and antibiotic-induced ototoxicity [17]. It has also been reported that miR-34a can serve as a potential biomarker to evaluate CDDP-related nephrotoxicity [18]. However, the role of miR-34a in cisplatin-induced ototoxicity remains unclear.

Mitochondria are highly dynamic organelles in eukaryotic cells that regularly fuse and divide themselves to maintain a balance, known as mitochondrial dynamics [19]. An abnormal balance between mitochondrial fission and fusion has been linked to various diseases, including cardiac diseases, neurologic diseases, cancer, and diabetes [20]. Mitochondrial dynamics participate in the oxidative stress response. It is well known that mitochondria are the main sources of ROS, the release of ROS causes further damage to mitochondrial components, and ROS-induced oxidative stress is involved in cochlear damage [21, 22]. Abnormal mitochondria can be eliminated through mitophagy. Dynamin-related protein 1 (Drp1), a GTPase enzyme, is an essential mediator of mitochondrial fission [23] to initiate mitophagy. *Yoshiyuki Ikeda. *et al. demonstrated that inhibition of mitophagy was caused by downregulation of Drp1, leading to mitochondrial accumulation [24].

According to the TargetScan database and the literature [25], DRP-1 is a target gene of miR-34a. To date, the role of miR-34a/DRP1 in cisplatin-induced ototoxicity has not been elucidated. The present study investigated the effect of miR-34a/DRP1 on mitophagy in the process of cisplatin-induced ototoxicity and the change in mitochondrial function to elucidate the possible mechanisms of cisplatin-induced ototoxicity.

## Materials and methods

### Materials

Cisplatin (CDDP, Selleck, S1166, USA), DRP1(immunoway, YT1414, USA), LC3B (ABclonal, A19665, China), β-actin（cell signalingtechnology, #4970, USA), Lipofectamine™ 3000 Transfection Reagent (Invitrogen, L3000001, USA), Myosin VIIa (Abcam, ab150386, England), BCA protein assay kit (Beyotime, cx00098, China), CCK8 ( APExBio, K1018, USA), JC-1 (Beyotime, C2006, China), DCFH-DA (Beyotime, S0033S, China), miR-34amimic/inhibitor/control (Ruibo, PA20201227003, China), and DAPI (Solarbio, C0065-10, China).

### Animals and cisplatin ototoxicity model

A total of 40 male C57BL/6 mice (18–20 g, 6 weeks old) were purchased from Guangdong Yaokang Biotechnology Company (Foshan, China). The mice were housed in the Animal Center of Jinan University at 23 ± 2 °C and 50–60% relative humidity with a 12 h light/dark cycle and free access to food and water. After adapting to the environment for 10 days and ABR measurement, the mice were randomly divided into a control group (*n* = 20) and a cisplatin group (*n* = 20). Three cycles of the cisplatin administration regimen were used according to previous studies to simulate the clinical administration of cisplatin to establish the ototoxicity model [26]. Briefly, cisplatin was dissolved in saline solution at a concentration of 1 mg/mL. The mice in the cisplatin groups were received 3.0 mg/kg cisplatin once daily (intraperitoneal injection) for 4 days, followed by 10 days for recovery as a cycle for a total of three cycles. The control groups were injected with normal saline (3 mg/kg.d) on the same schedule. All procedures of animal experiments were approved by the Committee on the Ethics of Animal Care and Use of Jinan University (Guangzhou, China, Permit NO. IACUC-20210426–02). All animals received research according to the criteria outlined in the “Guide for the Care and Use of Laboratory Animals” prepared by the National Academy of Sciences and published by the National Institutes of Health. All methods were reported in accordance with ARRIVE guidelines.

### Auditory Brainstem Response (ABR)

The mice were anesthetized using a mixture of ketamine (100 mg/kg) and xylazine (10 mg/kg). ABR testing was measured using Tucker-Davis Technologies (TDT system III, Alachua, FL, USA) 3 days before and at the end of cisplatin administration. Three subcutaneous needle electrodes were inserted at the vertex (active), under the pinna of the left ear (reference), and in the middle of the back (ground). The earphone was placed on the left auricle of the mice, and the sound stimuli were presented directly into the ear canal in the acoustic shielding room. The auditory waveforms within 10 ms (ms) were recorded after tone bursts with a 1 ms rise/fall time at frequencies of 8, 16, and 32 kHz. The average response to 1000 stimuli was obtained by reducing the sound intensity at 5 dB intervals from 100 to 0 dB SPL. The ABR threshold was defined as the lowest stimulation intensity that produced a replicable waveform response.

### Tissue preparation

At the end of 3 cycles of cisplatin administration, the deeply anesthetized mice were sacrificed by cervical dislocation after ABR detection and then decapitated, and the cochlea were collected. The temporal bones were washed with fresh ice-cold 4% PBS and then placed into a 30 mm diameter Petri dish containing fresh ice-cold 4% PBS. Under a dissection microscope, fine forceps were used to remove the stapes and tissue. The volute was scanned from the oval window parallel to the spiral of the basilar membrane using Venus scissors, and then a fracture line was cut from the bottom to the apical turn along with the spiral plane at the edge of the volute. The volute was gently removed with a fine forceps and needle, and the basilar membrane tissue was immediately placed into a centrifuge tube, snap frozen in liquid nitrogen, and stored at -80 °C for subsequent RNA or protein extraction. On the other hand, the cochlea was removed from the skull, the stapes was removed, a small hole was made in the apical turn of the cochlea, the round window was pierced, and 4% paraformaldehyde was perfused. Then, the cochlea was immersed in 4% paraformaldehyde overnight at 4 °C and decalcified in 10% sodium ethylenediaminetetraacetic acid for 48 h at room temperature on a shaker. The basilar membrane was dissected under a microscope for immunofluorescence staining.

### Hair cell counting

The basilar membrane samples were permeabilized with 2.5% Triton X-100 in 1X PBS for 15 min at room temperature on a shaker. Then, the specimens were washed 3 times with PBS and blocked in 10% goat serum solution for 1 h at room temperature. After washing with PBS three times, cochlear sections were incubated with phalloidin (1:200) for 2 h at room temperature in the dark, counterstained with DAPI for 8 min and washed three times with PBS. The samples were mounted on glass slides in 10 µl anti-fluorescence quenching agent. Hair cells were visualized using an Olympus BX63 fluorescence microscope from the apex to the base of the cochlea, and then the outer hair cells were counted.

### HEI-OC1 cell culture

House Ear Institute-Organ of Corti 1 (HEI-OC1) auditory cells were obtained from Lin Baixin Medical Research Central. Cells were cultured in Dulbecco’s modified Eagle’s medium (Gibco, USA) containing 10% fetal bovine serum (Gibco, USA) without antibiotics in a 33 °C incubator supplemented with 5% CO2 in air.

### Cell transfection

miR34a mimic or inhibitor and negative control were purchased from Ruibo Biology Technology Company (Ruibo, Guangzhou, China). HEI-OC1 cells were plated into 6-well plates at a density of 1.5 × 10^5^/well. Cells were grown to 50% confluence and then transfected with 5 µM miR34a mimic or 10 µM inhibitor using serum-free Opti-MEM (Gibco, USA) and Lipofectamine 3000 transfection agent (Invitrogen, USA) following the manufacturer’s instructions. Negative controls were generated using mimic or inhibitor control with the same procedure. Cells were incubated with the transfection mixture for 8 h at 37 °C, then replaced with normal DMEM and further incubated for 40 h.

### CCK-8 cell viability analysis

Cells were seeded in 96-well plates at a density of 5 × 10^3^ cells per well overnight. They were treated with various concentrations (10, 20, 30, and 40 µM) of cisplatin for different times (8, 16, 24, and 48 h) and 20 µM cisplatin for 24 h following transfection for 48 h. Cell viability was detected using Cell Counting Kit-8 (CCK-8) according to the manufacturer’s protocol. At the indicated time, 10 µl/well CCK-8 reagent was added and then incubated at 37 °C for 2.5 h in the dark. A microplate reader (Thermo Fisher Scientific, USA) was used to detect the absorbance at 450 nm.

### Intracellular ROS level detection assay

Intracellular ROS levels were detected by a Reactive Oxygen Species Assay Kit/DCFH-DA (2′,7′-Dichlorofluorescin diacetate) (Beyotime, S0033S, China), a fluorescent probe for living cells used according to the manufacturer’s protocols. The FACS Calibur system (BD Biosciences) was used to measure the green fluorescence intensity.

### Mitochondrial membrane potential (MMP) assay

Mitochondrial membrane potential assay kit with JC-1 was used to measure the MMP following the manufacturer’s instructions. JC-1 staining solution was diluted at a ratio of 1:1000 to the working concentration. After treatment with 20 µM cisplatin for 24 h, the cells were harvested, and then 1 mL of JC-1 working solution was added and incubated in the incubator at 37 °C for 20 min in the dark. Then, the cells were washed and analyzed using a FACS Calibur flow cytometer (Becton Dickinson, USA Bioscience).

### ATP content analysis

An Enhanced ATP Assay Kit was used according to the manufacturer’s protocols. The chemiluminescence intensity was measured by a SpectraMax M5 microplate reader (Molecular Devices)**.** The concentration of ATP in the sample was calculated according to the standard curve. The protein concentration was measured with the BCA protein quantification kit and normalized to nmol/mg.

### Real-time polymerase chain reaction (RT‒PCR)

Total RNA was extracted with an EZ-press RNA Purification Kit (EZBioscience, USA). Total RNA was reverse transcribed to cDNA using the Color Reverse Transcription Kit (EZBioscience, USA) following the manufacturer’s instructions. Reverse transcription was performed at 42 °C for 15 min and 95 °C for 30 s.

MiR34a expression was measured with Color SYBR Green qPCR Master Mix (EZBioscience, USA) by using the Roche LightCycler96 Real-Time PCR system (Roche Applied Science, Rotkreuz, Switzerland). The amplification program was 40 cycles of denaturing at 95 °C for 10 s, annealing at 60 °C for 30 s, and extension at 60 °C for 30 s. MiR-34a and U6 were purchased from RiboBio (Guangzhou, China). The sequences of specific primers were used as follows:

miR-34a forward:5’-ACACTCCAGCTGGGTGGCAGTGTCTTAGCTGGT-3’,

Reverse:5’-CTCAACTGGTGTCGTGGA-3’;U6forward:5’-GCTTCGGCAGCACATATACTAA-3’, reverse: 5’-AACGCTTCACGAATTTGCGT-3’. The expression level of miR-34a was defined from the Ct. U6 was used as an endogenous control. The 2^−ΔΔt^ method was used for relative quantification after normalization.

### Protein extraction and Western blot analysis

Protein was extracted from HEI-OC1 cells and cochlear tissue according to the manufacturer’s instructions. Protein samples (20 µg) were loaded in a 12.5% SDS‒PAGE gel and transferred to polyvinylidene fluoride membranes (Millipore, Burlington, MA, USA), followed by blocking with 5% nonfat milk in TBST buffer at room temperature for 1 h. The membrane was cut according to the target protein and then hybridized with the following primary antibody cocktail: anti-DRP1 (1:1000, immunoway), anti-LC3B (1:1000, ABclonal) and anti-β actin.

(1:2000, Cell Signaling Technology) at 4 °C overnight on a shaker. The strips were washed 3 times in 0.05% TBST for 10 min each time before incubation with HRP-conjugated anti-rabbit secondary antibody (Proteintech, 1:3000) for 1 h at room temperature. The protein intensity value was normalized by comparison with β-actin using ImageJ software (U.S. National Institutes of Health (NIH), Bethesda, MD, USA).

### Statistical analysis

The results were presented as data from at least three independent experiments and expressed as the mean ± S.E. (standard error of the mean). Statistical analysis was performed using two-way ANOVA with Duncan’s test or t test. SPSS 21.0 was used for statistical analyses. A value of *p* < 0.05 was considered significant.

## Results

### Cisplatin caused ototoxicity in C57BL/6 mice

**Fig. 1:**
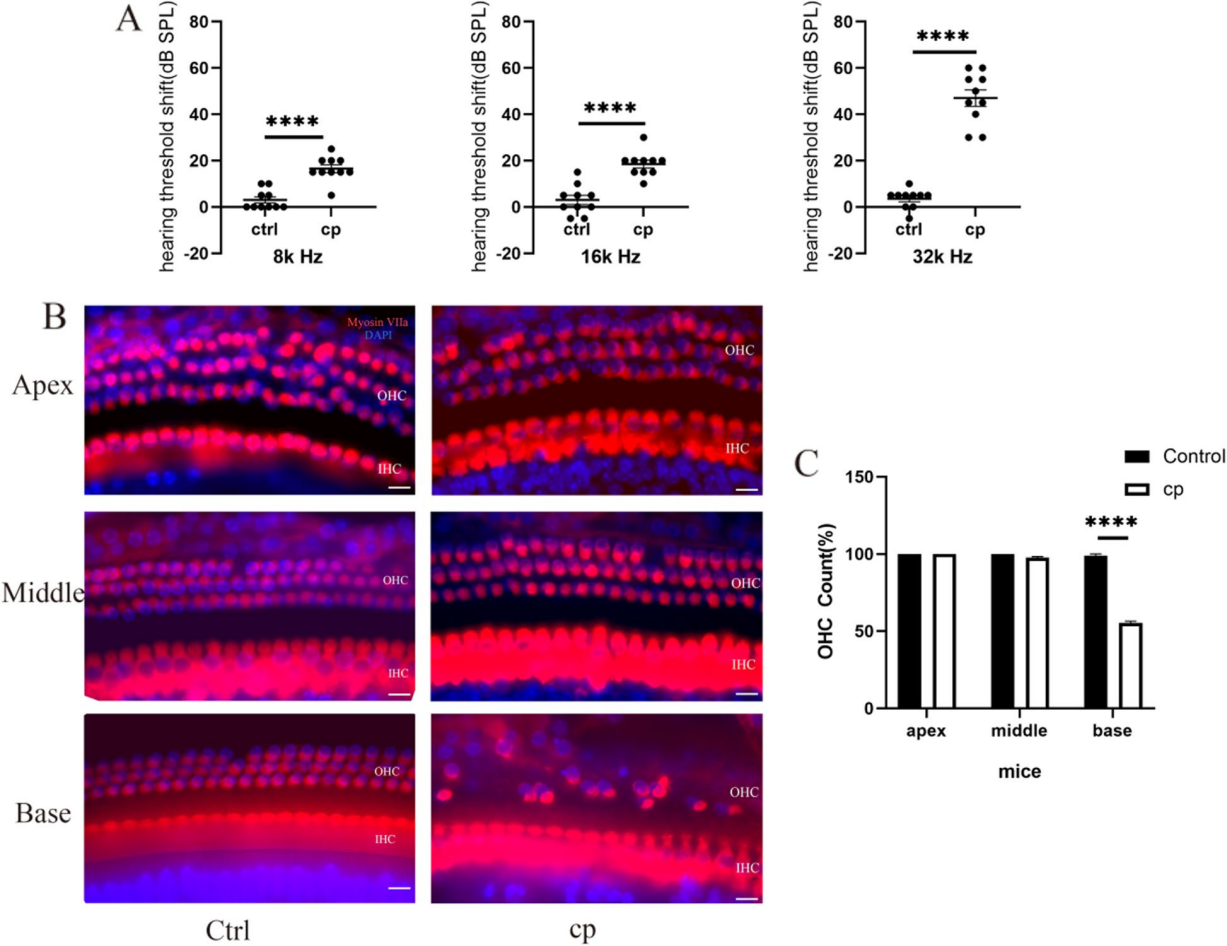
Three cycles of cisplatin treatment caused ototoxicity in C57BL/6 mice. **A** Hearing threshold shifts were observed in C57BL/6 mice at 8, 16 and 32 kHz. *N* = 10 per group. **B** Immunofluorescent staining (myosin VIIa) of the basilar membrane from a representative cochlear section.C. Outer hair cell counting obtained from five independent cochlear dissections at the apical, middle and basal turns. Scale bar = 20 µM. *n* = 5, ****P < 0.0001

The 3 cycles of cisplatin administration were performed according to previous studies [26]. ABR testing was used to evaluate the hearing level of C57BL/6 mice. The cisplatin group resulted in a greater hearing threshold shift than the control group, particularly at high frequency. The mean hearing threshold shift of the cisplatin group was 16.5 dB ± 5.29 at 8 kHz, 18.5 ± 5.29 dB at 16 kHz and 43 ± 18.59 dB at 32 kHz compared to 2.08 ± 4.5 dB at 8 kHz, 2.92 ± 5.82 dB at 16 kHz and 4.58 ± 4.98 dB at 32 kHz in the control group. The difference between the two groups was statistically significant at each frequency, but hearing loss was most significant at the high frequency of 32 kHz. (Fig. 1A, n = 10, *p* < 0.0001).

We dissected the cochlea to observe the morphological changes in the basilar membrane and identified the localization of hair cell loss using immunofluorescence staining. The results showed that 1 row of inner hair cells (IHCs) and 3 rows of outer hair cells (OHCs) were arranged neatly, without missing from the apical to basal cochlear turns in the control group (Fig. 1B). Thus, in C57BL/6 mice treated with cisplatin, the missing outer hair cells were mainly located at the basal turn (Fig. 1B), and the survival rate of outer hair cells was 54.98 ± 1.9% (Fig. 1C, *n* = 5, *P* < 0.05). The 3 cycles of cisplatin administration in C57BL/6 mice were similar to the clinical medication regimen. The results of decreased hearing and hair cell loss were consistent with previous studies [26] in the mouse model and indicated that cisplatin caused ototoxicity in C57BL/6 mice.

### The expression of miR34a/DRP1 in the cochlea of C57BL/6 mice after 3 cycles of cisplatin treatment

**Fig. 2:**
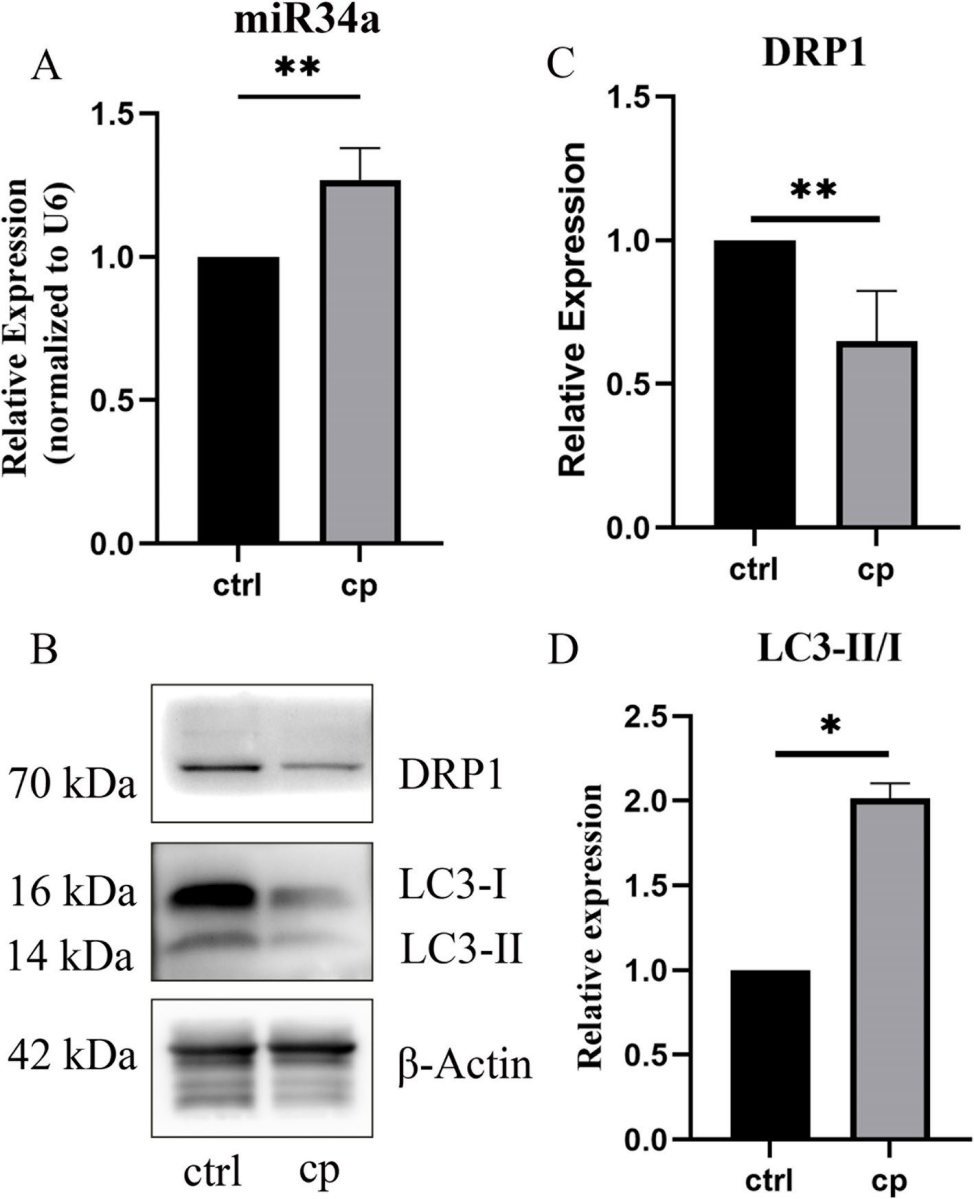
The expression of miR-34a and DRP1 in C57BL/6 mice treated with cisplatin. **A** RT‒PCR showed that the expression of miR-34a was increased in the cisplatin group. **B-D** The levels of DRP1 and LC3II/I were detected using Western blotting in C57BL/6 mice. **B** Representative Western blot analysis of DRP1 and LC3-II/I. **C-D** Relative expression of DRP1 and LC3-II/I. Data are presented as the mean ± SEM of three independent experiments. *p < 0.05, **p < 0.01

In this study, we found that miR34a expression was significantly upregulated in mice treated with cisplatin via RT‒PCR (Fig. 2A, *P* < 0.05). Western blot analysis showed that DRP1 protein levels were decreased, whereas LC3-II/I levels were elevated in the cisplatin group (Fig. 2B, C, D, *P* < 0.05). Thus, we speculated that miR-34a/DRP-1 may be involved in the process of cisplatin-induced ototoxicity in C57BL/6 mice.

#### Cisplatin induced cytotoxicity via mitochondrial dysfunction in HEI-OC1 cells

**Fig. 3:**
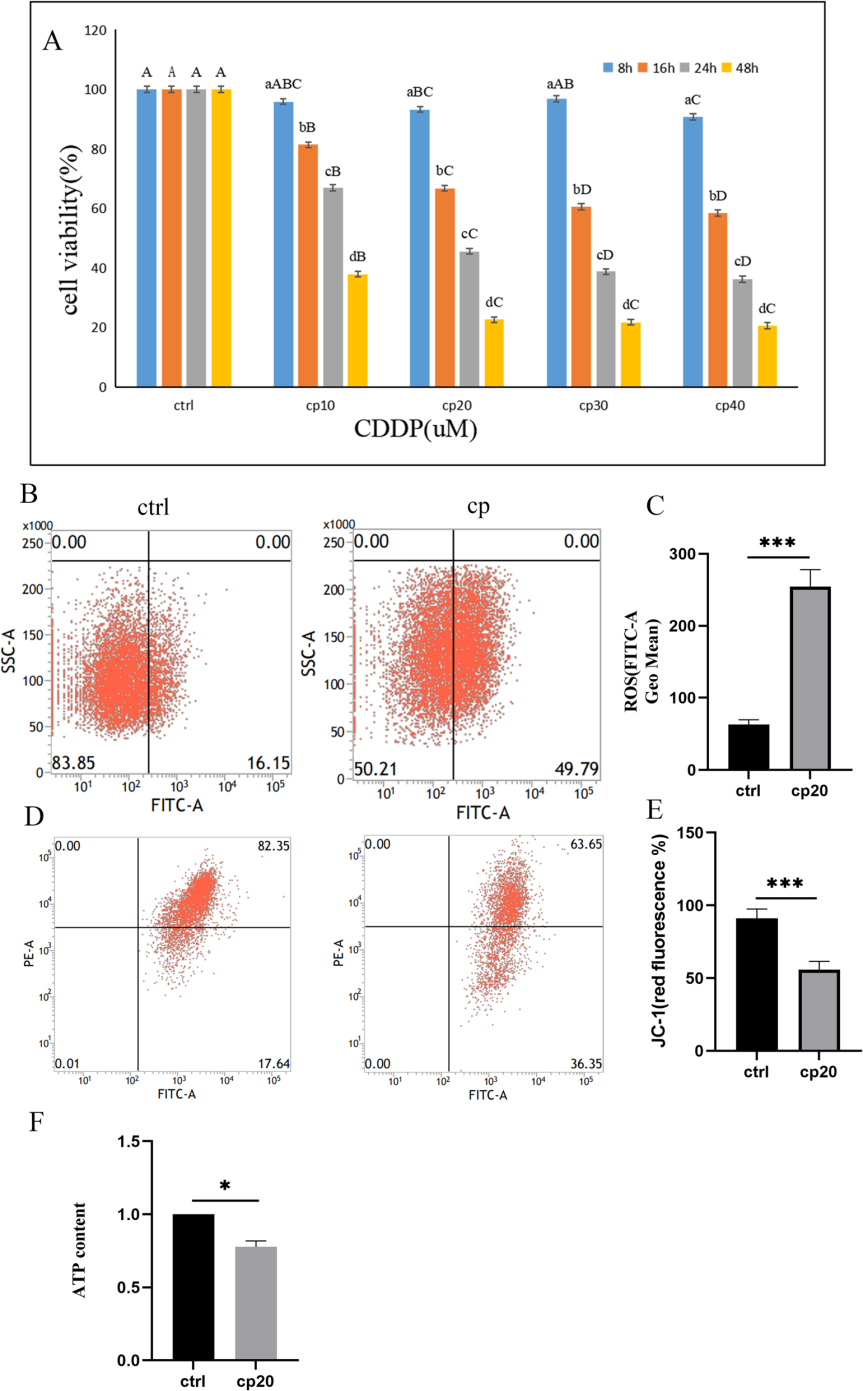
Cisplatin reduced cell viability and affected mitochondrial function in HEI-OC1 cells. **A** Cell viability was determined using the CCK-8 assay. HEI-OC1 cells were treated with various concentrations (0, 10, 20, 30, 40 µM) of cisplatin for 8, 16, 24, and 48 h. Cell viability decreased with cisplatin treatment in a time-dose-dependent manner. **B**, **C** FITC fluorescence intensity was measured using flow cytometry. Cisplatin exposure increased ROS levels. **D**, **E**, **F** Cisplatin treatment impaired mitochondrial function. Data are presented as the mean ± SEM of three independent experiments. *p < 0.05;**p < 0.01;***p < 0.001

HEI-OC1 cells were treated with various concentrations of cisplatin (10, 20, 30, and 40 µM), and cell viability was detected at 8, 16, 24, and 48 h after exposure. The CCK-8 assay indicated that cisplatin exposure induced the cytotoxicity of HEI-OC1 cells in a dose- and time-dependent manner (Fig. 3A). The cell viability was approximately 45.2% in HEI-OC1 cells treated with 20 µM cisplatin for 24 h. Therefore, we chose 20 µM cisplatin for 24 h as the exposure concentration and time for subsequent experiments.

ROS formation is an important marker of oxidative stress, and mitochondria are the main sites of oxidative stress in cells. A previous study revealed that cisplatin application increased the generation of ROS [27]. The ROS level in HEI-OC1 cells after 20 µM cisplatin treatment for 24 h was assessed by DCFH-DA staining. The FACS results showed that the green fluorescence intensity was significantly increased after cisplatin treatment (Fig. 3B, C).

Mitochondrial membrane potential (MMP, ∆Ψm) is an indicator of mitochondrial integrity and bioenergetic function [28]. JC-1 is a widely used fluorescent probe in the detection of MMP. The red and green fluorescence intensities were detected by flow cytometry. The red fluorescence intensity was significantly decreased in the cisplatin group (Fig. 3D, E). Meanwhile, the ATP content also declined in the cisplatin group (Fig. 3F).

These data demonstrated that cisplatin induced cytotoxicity in HEI-OC1 cells through mitochondrial dysfunction.

#### The expression of miR34a/DRP1 in HEI-OC1 cells after cisplatin treatment

**Fig. 4:**
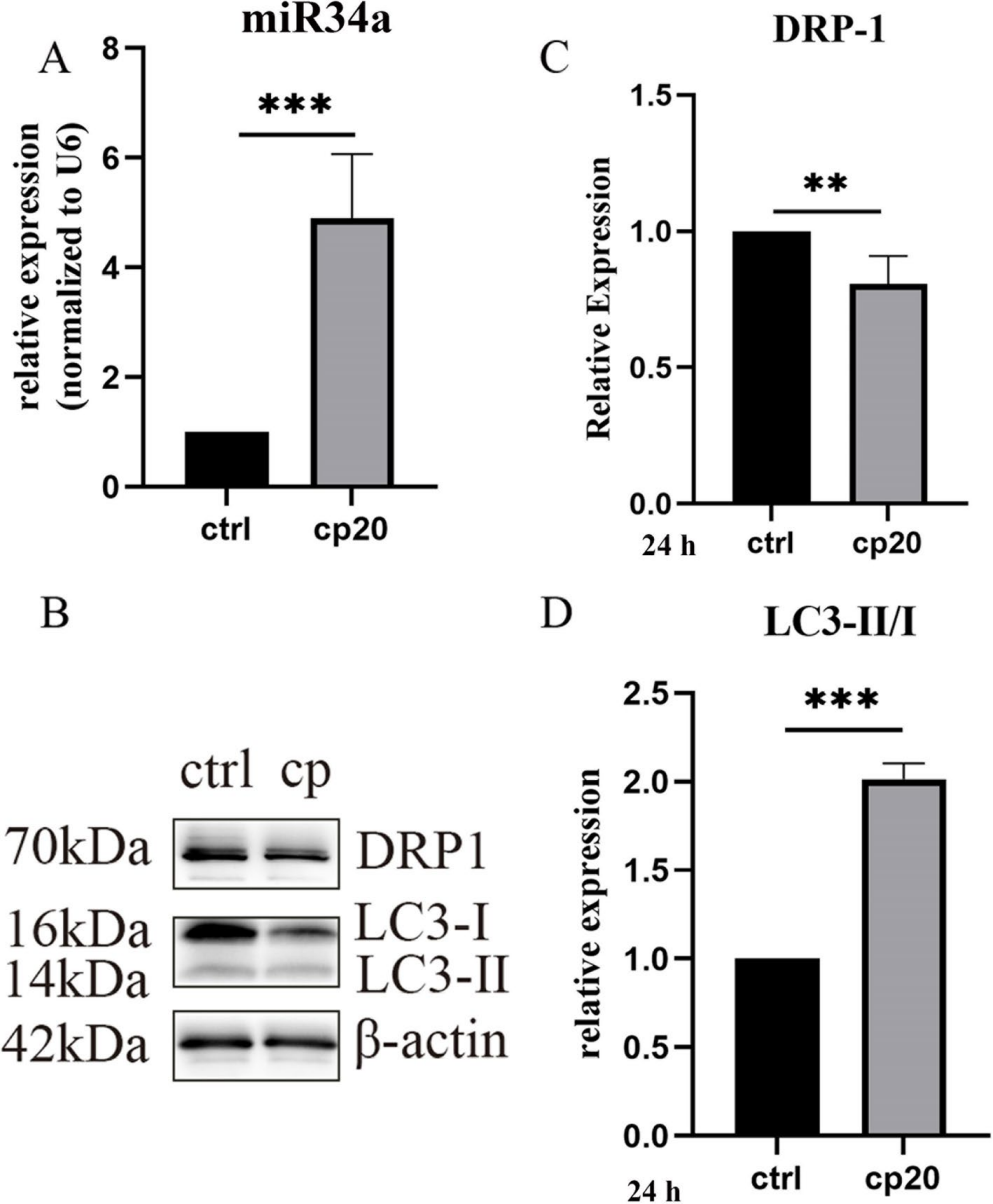
The expression of miR-34a and DRP1 in HEI-OC1 cells treated with 20 µM cisplatin for 24 h. **A** RT‒PCR showed that the expression of miR-34a was increased in the cisplatin group. **B**-**D** The levels of DRP1 and LC3II/I were detected using Western blotting in HEI-OC1 cells. **B** Representative Western blot analysis of DRP1 and LC3-II/I. **C**, **D** Relative expression of DRP1 and LC3-II/I. Data are presented as the mean ± SEM of three independent experiments. *p < 0.05, **p < 0.01

In HEI-OC1 cells treated with 20 µM cisplatin for 24 h, RT‒PCR showed that miR34a expression was significantly upregulated (Fig. 4A), DRP1 protein expression was decreased, and LC3-II/I levels were elevated, as shown by western blot analysis (Fig. 4B-D), in line with the in vivo results. Based on the above data, we speculated that miR-34a/DRP-1 may play an important role in cisplatin-induced ototoxicity via mitochondrial dysfunction.Next, we investigated the effect of miR-34a/DRP1 on mitophagy and the underlying mechanism.

#### miR-34a modulated DRP1 expression and mitophagy

**Fig. 5:**
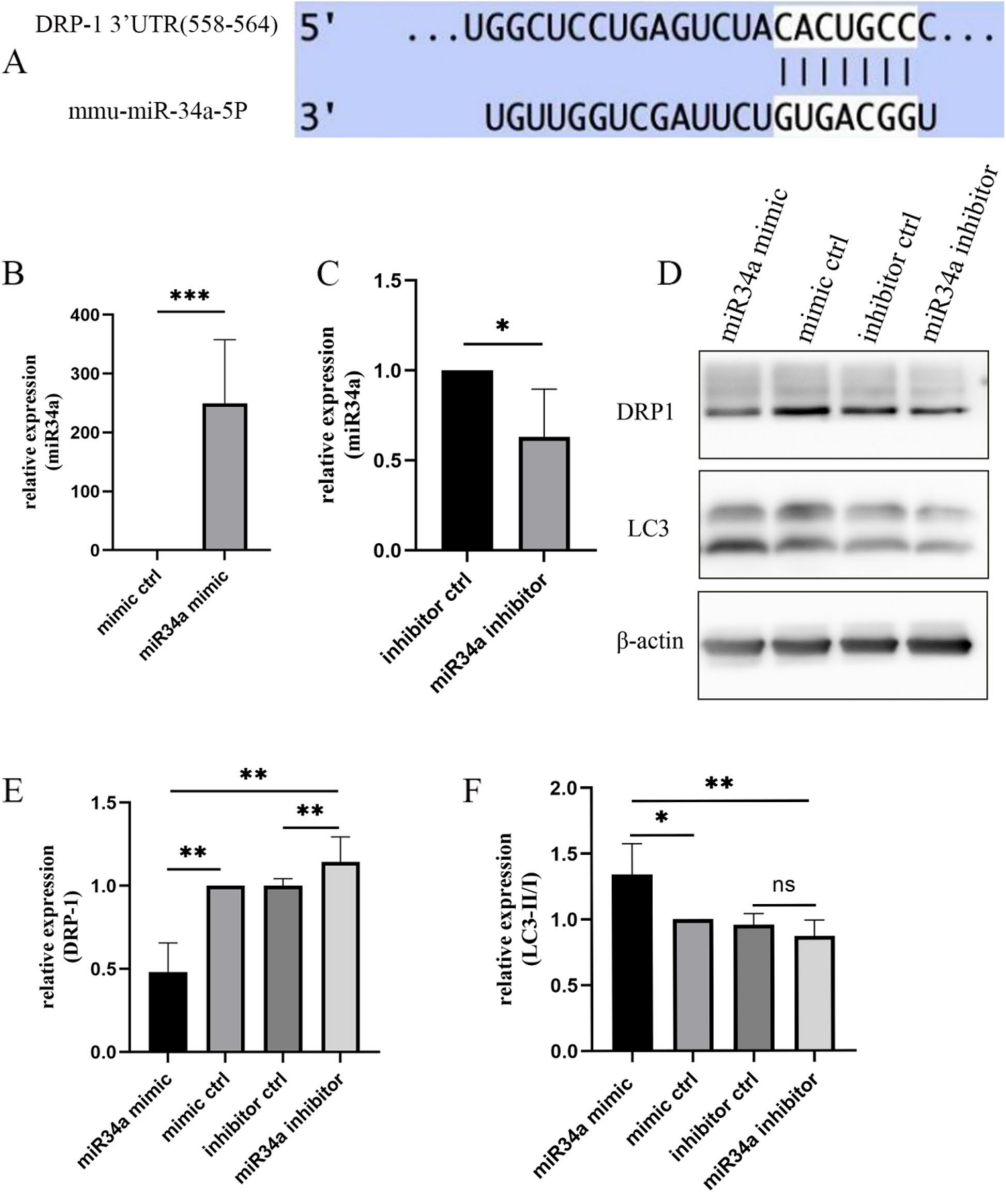
MiR-34a modulated DRP1 expression and mitophagy in HEI-OC1 cells. **A** The putative binding site of miR-34a-5p on the 3’-UTR of DRP1 as predicted in the TargetScan database. **B**, **C** qRT‒PCR showed the level of miR34a in HEI-OC1 cells transfected with miR-34a mimic inhibitor or control. **D**-**F** Western blot analysis showed DRP1 and LC3-II/I expression in HEI-OC1 cells after miR34a mimic, inhibitor or control transfection

By searching the TargetScan database and the literature [25], we found that DRP1 might be a target protein of miR-34a (Fig. 5A). Modulation of miR-34a in HEI-OC1 cells further investigated the effect of miR-34a on DRP1 expression and mitophagy.

RT‒PCR showed the transfection effect (Fig. 5B, C). Western blot analysis showed that DRP1 decreased and LC3-II/I expression increased in HEI-OC1 cells overexpressing miR-34a; however, the results were reversed in HEI-OC1 cells treated with the miR-34a inhibitor (Fig. 5D-F).

#### miR-34a mediated cisplatin-induced ototoxicity via the regulation of mitochondrial function

**Fig. 6:**
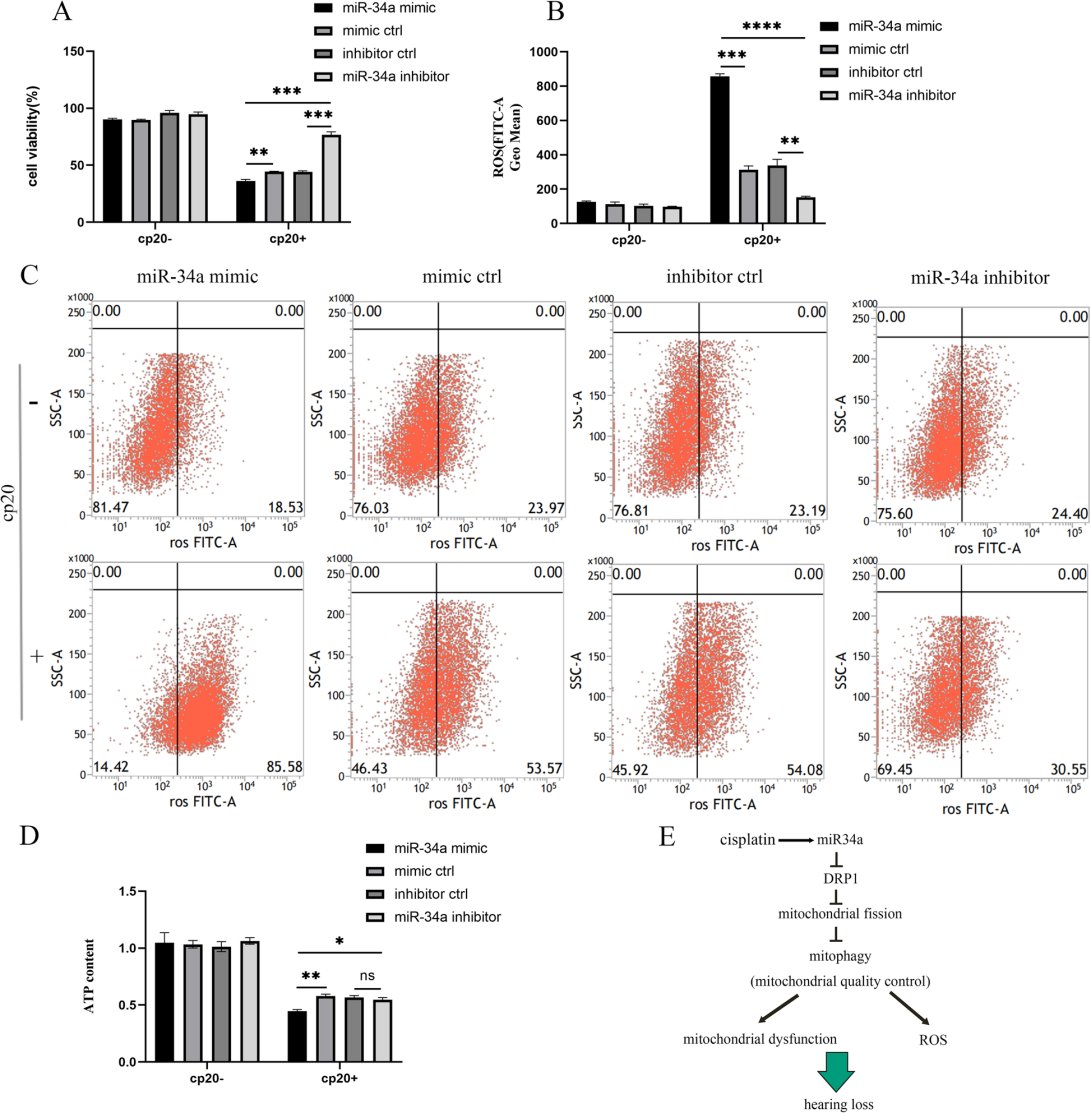
Role of miR-34a/DRP1 in cisplatin-induced ototoxicity in HEI-OC1 cells. Cells were transfected with miR-34a mimic or inhibitor and negative control miRNA and then incubated with 20 µM cisplatin for 24 h. **A** Cell viability was detected by CCK8 assay. **B**, **C** The green fluorescence intensity of ROS detected by flow cytometry. **D** ATP content was detected by chemiluminescence. **E** Model summarizing the relationship between miR-34a/DRP1 and mitophagy in cisplatin-induced ototoxicity. Data are presented as the mean ± SEM of three independent experiments. *p < 0.05;**p < 0.01;***p < 0.001;****p < 0.0001

To further investigate the effect of miR-34a on cisplatin-induced ototoxicity, HEI-OC1 cells were transfected with miR-34a mimic or inhibitor and the corresponding negative control and then exposed to 20 µM cisplatin for 24 h, and cell viability, ROS level and ATP content were detected (Fig. 6). Compared with the negative control miRNA, miR-34a overexpression resulted in a decrease in cell viability and ATP content and an increase in ROS levels in HEI-OC1 cells. Therefore, we speculated that the increase in miR-34a levels might aggravate ototoxicity by enhancing oxidative stress and mitochondrial dysfunction after cisplatin exposure. Furthermore, inhibiting miR-34a exerted the opposite tendency, significantly improving cell viability and ATP content and decreasing ROS levels relative to the miR-34a mimic group after cisplatin treatment, indicating that decreased miR-34a can alleviate ototoxicity by reducing oxidative stress and improving mitochondrial function. Because the MMP of the negative group decreased too much after JC-1 staining, the MMP result was not included in this part.

## Discussion

Currently, cisplatin-induced ototoxicity is a major obstacle that limits the maximum efficacy for tumor patients. Previous studies have demonstrated that miRNAs are closely associated with hearing loss and are considered promising therapeutic targets [29]. A recent study reported that mitophagy protected HEI-OC1 cells against cisplatin-induced ototoxicity [30], but the precise molecular mechanism remains to be further studied. In the present study, we investigated whether miR-34a/DRP1-mediated mitophagy contributed to cisplatin-induced ototoxicity and sought to determine the underlying mechanism.

Three cycles of cisplatin treatment resulted in increased miR-34a and decreased DRP1 in the cochlea of C57BL/6 mice, accompanied by significant hearing threshold elevation and outer hair cell loss. Meanwhile, cisplatin caused ototoxic damage and mitochondrial dysfunction in HEI-OC1 cells, and the changes in miR-34a and DRP-1 expression were consistent with the results in C57BL/6 mice. Based on these results, we considered that miR-34a and DRP-1 were involved in the process of cisplatin-induced ototoxicity with mitochondrial dysfunction and that autophagy may be activated in some way. Current studies on the role of autophagy in cisplatin-induced ototoxicity are contradictory [31, 32]. In this study, we focused on the role of mitophagy, a selective autophagy that plays an important role in removing damaged mitochondria and maintaining the dynamic stability of the mitochondrial network, thereby protecting the cell [33].

Mitochondrial homeostasis is maintained through a dynamic balance of fusion and fission [34]. DRP-1 is a cytosolic GTPase that regulates mitochondrial fission, which is important for mitochondrial renewal, proliferation, and redistribution to maintain mitochondrial morphology, number and functionality [35, 36]. By searching the TargetScan database and the literature, we found that DRP-1 might be a target protein of miR-34a. Herein, we further verified the effect of miR-34a on DRP-1 expression and found that miR-34a overexpression led to decreased DRP-1 expression and an increased LC3 II/I ratio in HEI-OC1 cells. Nevertheless, inhibiting miR-34a expression can reverse these results. Therefore, we proposed that miR-34a contributed to cisplatin-induced ototoxicity by negatively regulating the expression of DRP-1 and damaging mitophagy.

Subsequently, we investigated the effect of miR-34a regulation on mitochondrial function during cisplatin treatment in HEI-OC1 cells. We found that cell viability and mitochondrial function decreased in HEI-OC1 cells overexpressing miR-34a compared with the negative control group after cisplatin exposure. In addition,

inhibition of miR-34a expression could reduce the damage to cell viability and mitochondrial function after cisplatin exposure. The results indicated that modulating miR-34a expression can improve ototoxic damage by regulating mitochondrial function.

Taken together, the results of this study revealed that miR-34a/DRP1 played an important role in cisplatin-induced ototoxicity and was probably related to abnormal mitophagy. We speculated that the increase in miR-34a expression resulted in a decrease in DRP1 expression and led to abnormal mitophagy, ultimately causing ototoxicity during cisplatin treatment. Furthermore, inhibiting miR-34a expression alleviated cisplatin-induced ototoxicity, which was probably linked to the improvement of DRP1-mediated mitophagy, thus removing abnormal mitochondria and improving mitochondrial function. Therefore, miR-34a/DRP-mediated mitophagy may be a novel target for investigating the treatment and protection of cisplatin-induced ototoxicity.

## Limitations

There are still limitations that need to be further studied regarding this research. We can try to regulate the expression of miR-34a in cochlear explants or use transgenic mice to determine the effect of miR-34a/DRP1 on cisplatin-induced ototoxicity, and the results would be more convincing. Furthermore, direct regulation of DRP1 expression determined the role of DRP1 in the development of cisplatin ototoxicity.

## Data Availability

All data used to support the findings of this study are included within the article.
